# Informal Caregiving in Pakistan: Role of Women Family Caregivers in Older People's Health

**DOI:** 10.1155/jare/2378593

**Published:** 2025-11-04

**Authors:** Sumera Saeed Akhtar, Susan Heydon, Nadeem Irfan Bukhari

**Affiliations:** ^1^Department of General Practice and Rural Health, University of Otago, Dunedin, New Zealand; ^2^School of Pharmacy, Division of Health Sciences, University of Otago, Dunedin, New Zealand; ^3^Punjab University College of Pharmacy, University of the Punjab, Lahore, Pakistan

**Keywords:** access to healthcare services, caregivers, decision-making, medicine practices, older people

## Abstract

**Introduction:**

This study explores the role of Pakistani women caregivers in older people's decision-making regarding access to healthcare services and medicine practices, including attitudes towards medicine adherence and self-medication.

**Methods:**

Data were collected from 52 women who cared for older people using focus group discussions and semistructured interviews. The study was conducted in the rural and urban regions of Sargodha District, Punjab.

**Results:**

Informal women caregivers play a pronounced role in decision-making about healthcare access and medicine-taking practices for older people. Treating illnesses at home with allopathic medicines is a common practice in both urban and rural settings. Caregivers were unaware of the risk associated with a delay in health-seeking and self-medication.

**Conclusion:**

Findings suggest that educational interventions and coaching should be provided to improve family caregivers' knowledge so that they can contribute to better health outcomes for older people.

## 1. Introduction

According to the World Health Organization (2018), the proportion of the global population aged 60 and older is expected to double from 12% to 22% between 2015 and 2050. By mid-century, around 80% of the world's older population will live in low- and middle-income countries (LMICs). This demographic shift has brought the issue of support for older people to the forefront of contemporary social and health policy debates, especially in developing nations. These countries are experiencing a rapid increase in their ageing populations, a transformation often occurring within a single generation [1]. Pakistan, a South Asian country, with an elderly population of 11.3 million (out of a total population exceeding 200 million), is expected to rise to 43.3 million by 2050 [2].

Informal caregiving is deeply ingrained in Pakistan's sociocultural traditions that stress the family's role in supporting the elderly. Religious principles further strengthen this practice, instilling a moral duty in children to look after their ageing parents. Similar patterns are observed in other collectivist societies, where cultural norms emphasise cooperation, responsibility, interdependence and respect across generations [3]. Such cultures are often shaped by the ideals of familism; older adults are held in high esteem, and children are expected to care for their parents selflessly, placing their parents' needs above their own [4]. It is common in Pakistan to practice a joint family system based on the values and respect of older people [5, 6].

No formal care system exists under the governance of public or private bodies. Recently, the government introduced homes for older people. However, these are very few, will benefit less than 1% of the older population and are only in three big cities. Informal or family caregivers are unpaid carers who are usually family members, particularly women [7]. Studies have shown the economic value of such informal caregiving to be hundreds of billions of dollars [8]. Informal caregivers play a crucial role among family members, particularly in Pakistani culture and society, where strong family ties and bonding are deeply ingrained social norms. Family members can assist in activities of daily living (ADLs), such as grooming, walking, eating/feeding and providing emotional support to the dependent individuals [9].

Caring for family members is considered predominantly a woman's responsibility [10]. This responsibility of caring within the family seats women at the interface between the family and the state [11]. They are well-practised at caring for their family responsibilities by the time they need to care for either a parent or a spouse. This caring woman's role is either self-directed, persuasive or under the influence of relatives [12]. The caregiver faces an overwhelming array of decisions during a patient's illness. The patient–family unit generally decides such as treatment options and finances [13, 14].

Medication management has been recognised as one of the key responsibilities undertaken by family caregivers in community settings [15, 16]. An online survey conducted in the USA revealed that 607 (36%) out of 1677 informal or family caregivers reported managing medications for the individuals under their care [17]. This role is critically important, as effective medication management by family caregivers has been shown to enhance health outcomes and reduce the likelihood of institutionalisation for care recipients [18]. Unlike many countries, Pakistan does not have a national Medicare or Pharmacare-type entitlement. Since the Eighteenth Constitutional Amendment in 2010, health planning and service delivery have been the responsibility of provinces, rather than the federal government [19]. Instead of universal entitlements, provinces participate in social health protection schemes, such as the *Sehat Sahulat* (health facility) and *Sehat* Card (health card) programmes, which mainly cover inpatient admissions at public or private hospitals [20]. However, routine outpatient department (OPD) care is generally not included in the above facilities. In practice, patients often have to pay out-of-pocket for OPD consultations, medicines and diagnostics, including at public facilities, unless they are admitted under a covered package. Provincial policies vary, and overall, the financing context remains heavily reliant on out-of-pocket payments; in 2021 and 2022, 52.6% of health spending went through the private sector, and 89% of that private spending was household out-of-pocket [21]. This pattern helps explain why older adults are hesitant to seek timely care for conditions that require frequent OPD visits and medicines.

A study conducted in Pakistan involving interviews with 921 respondents revealed that females particularly bear the burden of caregiving in terms of physical, emotional and financial stresses [22]. Other studies examined the barriers to medication adherence among older people with chronic illnesses [23–25]. However, the limited focus has been on exploring health-seeking practices and medication management for care receivers. To the best of our knowledge, no study has explored the role and involvement of Pakistani women as family caregivers in decision-making for health-seeking, utilising available healthcare and medication management of older people under their care in rural and urban settings. Focussed on the need to explore the involvement of women family caregivers in older people's healthcare practices in rural and urban settings, this study's findings could be generalised to countries with a similar culture of family systems, particularly in South Asian.

### 1.1. Ethical Approval

Ethical approval was obtained from the Human Ethics Committee, University of the Punjab, Lahore, Pakistan (#HEC/PUCP/1950).

## 2. Materials and Methods

### 2.1. Interview Guide

The research team developed an interview guide for this study, containing open-ended questions that explored the role of women caregivers in the healthcare practices of older people. This ensured consistency between participants in one-to-one interviews and face-to-face focus group discussions (FGDs). The validated interview guide was piloted on three women caregivers and amended accordingly where needed. However, the response in the pilot study was not included in the results.

### 2.2. Study Design

This exploratory qualitative study used inductive thematic analysis, guided by sensitising concepts and did not impose an a priori theory [26]. The study utilises one-on-one semi-structured interviews and FGDs as primary data sources. The research aimed to explore and gain a deeper understanding of the role played by women caregivers in the healthcare practices of older people [27, 28].

### 2.3. Participants

#### 2.3.1. Eligibility Criteria

Informal women caregivers aged 18 years or older who provided care for the elderly aged 60 and above requiring assistance in at least two ADLs, medicine-taking and healthcare-seeking practices. Participants who provided care for at least 3 years were recruited (both for FGDs and interviews). The participants caring for older people in urban and rural areas were primarily wives, unmarried or married daughters and daughters-in-law.

### 2.4. Study Setting and Recruitment

The study was conducted in the rural and urban regions of Sargodha District, Punjab, the hometown of S.S.A. In 2018, the district had a population of 3.69 million [29, 30]. Most of the population resides in rural areas, around 2.55 million, and 1.14 million in urban areas. The participants were recruited through several visits to the central city and nearby rural areas, where discussions with the people in the area's public school helped identify participants. A purposive sampling approach [31] was used to enhance recruitment. The saturation point criteria were used to conclude the data collection [32]. Participants were given the option to choose between one-to-one interviews and FGDs. The interviews were conducted at the place of participant choice (either their home or nearby public school), where they felt safe disclosing their experiences. A nearby public school room was hired to conduct FGDs. Recruitment started in November 2018 and continued through to March 2019.

### 2.5. Data Collection

S.S.A. conducted all the interviews and FGDs after training in qualitative research. The study purpose was explained at the recruitment stage of the FGDs and interviews, and an informal conversation about the project and the researcher's introduction occurred. S.S.A. read the information sheet and consent form in Urdu and provided an opportunity to ask questions. Following that, S.S.A. offered them options to consider whether they would continue to participate and their right to withdraw from the study at any time. Confidentiality and their right to remain anonymous were also explained in detail. All participants received an information sheet and signed consent forms before semi-structured interviews and FGDs, followed by verbal consent, which was recorded at the start of each interview. With the participants' permission, all discussions between the interviewer and the respondent were audio-recorded. Demographic information was collected before the start of formal FGDs and interviews with participants. For example, the caregivers of older people were asked about their age, education, occupation, relationship with the care receiver, age of the care receiver and occupation of the care receiver. The interview questions began broadly, in line with the established guidelines, and subsequent probing was used to elicit more in-depth and detailed responses. S.S.A. moderated all of the FGDs and took notes during and after interviews. Interviews and FGDs were carried out in Urdu. Data were recorded in different ways, such as through audio recordings of interviews and notes taken during the interviews. All the identifying information about the participants was removed, and transcripts were labelled with numbers. The one-on-one interview lasted 30–45 min, and FGDs lasted 90–120 min.

### 2.6. Data Analysis

For reliability in the analysis of the interviews, researchers independently reviewed all the interview data. S.S.A. transcribed each recorded interview in Urdu, and the transcripts were reviewed alongside field notes to gain a comprehensive understanding. A bilingual translator directly involved in the study translated all transcripts into English. Subsequently, the transcriptions and translations were double-checked by other investigators. The inductive thematic analysis approach, which involves six phases: familiarisation with the data, generation of initial codes, searching for themes, reviewing themes, defining and naming themes and producing the report, was used to analyse the data [33].

To become familiar with the data, S.S.A. reread all the transcripts several times and coded them. Relevant words, phrases and sentences indicating the study objectives were labelled, and relevant codes were assigned to them, generating initial codes. The coded data were then reduced and organised to draw final themes and subthemes through regular debriefing meetings of S.S.A., S.H. and N.I.B., which reached a consensus. All the transcripts were analysed using NVivo 12 Plus. The drawn themes and conclusions were repeatedly studied to confirm that they reflected the aims of the study. Cross-checking of the identified themes and conclusion was undertaken and discussed with the research team to confirm the aims of the study and to ensure data credibility.

## 3. Results

Based on the women caregivers' involvement and role, we identified three main categories: [34] healthcare seeking, [1] access to healthcare services and [2] medicine practices. Thematic subcategories cover various aspects of the main categories.

### 3.1. Demographic Characteristics of Women Caregivers

The sample consisted of 52 women caregivers for older people. Of these, 32 women caregivers participated in 4 FGDs, with 2 from each of Sargodha district's rural and urban areas. The FGDs for caregivers of older people in the rural Sargodha area had 15 participants (7 in one group and 8 in the other). In the urban areas, 17 women participated (9 in one group and 8 in the other). The details about the participants in FGDs are reported in Table 1.

Most of the FGD participants in the rural area were aged between 40 and 59 years, and 60–79 years in the urban area. Most of the care receivers' ages ranged from 80 to 89 years for both urban and rural samples. There was a remarkable difference between the education level of caregivers in the urban and rural areas, as more caregivers (urban) were well-educated than those in the rural sample. Most of the caregivers in the urban area were housewives compared to those in the rural area, where most were casual workers working with their families on farms. In rural areas, most caregivers of older people were daughters-in-law, while in the urban areas, they were wives.

There were 20 one-to-one semi-structured interviews; 10 each conducted in Sargodha's rural and urban suburbs. The information about the participants is summarised in Table 1. Most caregivers were 40–49 years old in rural areas compared to 60–79 years old in urban areas, which confirms that in rural areas, daughters-in-law usually take care of older people. However, in urban settings, wives take care of older husbands. As expected, the caregivers and care receivers in the urban areas were more educated than those in the rural areas.

### 3.2. Demographic Characteristics of Care Receivers

Among the 52 older people cared for by the participants, 9 were bedridden. Of these, 4 were from the village sample, and 5 were from an urban area. They depended entirely on the caregiver for all their needs, such as feeding, bathing and medicines. The remainder could walk but needed the assistance of caregivers to walk, feed and take medicine. Care receivers take medication for Alzheimer's, Parkinson's disease, dementia, diabetes, high blood pressure, arthritis, depression, osteoporosis, respiratory conditions, such as asthma and chronic obstructive pulmonary disease and chronic pain; similar results were found in both the rural and urban samples. Most of the care receivers were prescribed multiple medications by multiple healthcare providers. It was also found that in rural and urban areas, the women caregivers got help from other family members when living in a joint family system.

### 3.3. Healthcare Seeking

#### 3.3.1. Treating at Home

Overall, caregivers reported that older people under their care are treated using home remedies for minor ailments and avoid visiting healthcare providers for a few days. They also stated that if the patient was not relieved or the conditions worsened, they obtained medicine from the nearest dispensary (local healthcare centre). Most women caregivers said they took their older adults to the emergency department of a private or government hospital if the symptoms worsened. The decision to take them to the government hospital depends more on the ambulance service, which takes the patients to government hospitals. As the rural areas are far from the government hospitals, caregivers initially treated them at home. They sometimes took them to the dispenser (qualified health technicians) to get medicine for instant relief or for symptoms to subside.

“*When he has a fever, flu, or a cough, we usually take medicine from a dispenser near to us.*” (I1: Caregiver: Daughter, Urban)

*“Sometimes, she has abdominal pains and cramps. Maybe due to gas, she wants Pakhi [herbal powder mix for digestion] for that, so I give her that.”* (FGD 1: Caregiver: Daughter-in-law, Urban)

It was also observed that older care receivers with better cognitive ability self-identify their symptoms and get involved with their medicines and treatments. However, their reluctance may delay the doctor's visit up to a week or two after the onset of the initial symptoms.

#### 3.3.2. Decisions About Visiting a Doctor/Hospital

Mostly, women caregivers play the principal role in decision-making and informing other family members about the older person's condition. However, other family members, such as a son or the spouse of the older person, or sometimes the care receiver themselves, get involved in decision-making, depending on their cognitive ability. Nevertheless, the caregivers are involved in decision-making as they are more aware of the older person's health condition. The woman caregivers, such as the daughter-in-law, wife and daughter, accompany the older person to the hospital along with a male family member. In the rural and urban samples, some caregivers stated that the care receiver influenced the decision to access healthcare, as sometimes they insisted on visiting the doctor or nearby dispensary for their pains or unbearable health conditions. Sometimes, participants reported that their older person had become “stubborn” and wanted to avoid visiting doctors. Many factors influence caregivers' seeking health care for older people, including financial status, area of residence, transportation cost and eligibility for adequate health services, such as military hospitals.

*“My husband and I took her to the doctor. He usually remembers the instructions. After coming home, he told me about the instructions, such as when and how to give the medicine. I usually give her medicine, but she also remembers it from the tablet box.”* (I3: Caregiver: Daughter-in-law, Rural)

*“He was not well, was unable to breathe, eat and talk, his chest was blocked, but he was stubborn about going the doctor. We forcefully put him in a taxi as he was dragging his feet at the door.”* (FGD 1: Caregiver: Daughter, Urban)

### 3.4. Access to Healthcare Services

#### 3.4.1. Visiting Government or Private Hospital

Government hospitals in Pakistan provide subsidised health services to older people, but caregivers have reported preferring private hospitals. All the urban caregivers for older people said they have easy access to all the good private and government hospitals. It costs them a little, as most have their transport. All the rural caregivers said it took almost 45–60 min to visit any good private or government hospital in the city. It costs them a lot as most need transport and must arrange transport before the appointment. Most rural caregiver participants said that arranging transportation to visit the doctor doubled the cost as they had to take the older people with them and hire a taxi for a day.

“*My mother-in-law cannot travel as she cannot sit, so we have to give support while she is sitting. We have to rent the car for a whole day trip to the city for the doctor.*” (I6: Caregiver: Daughter-in-law, Rural)

Most caregivers mentioned that while visiting the doctor, they are usually accompanied by one male family member, such as the spouse or son of the care receiver.

*“My husband and I take our mother-in-law to the doctor as I take care of her medicines and food, so I have to be with them. So, we take a rickshaw as it takes thirty minutes from here to the doctor's place.”* (FGD 1: Caregiver: Daughter-in-law, Urban)

Most participants said they sometimes travel to other far cities for specific consultations and surgeries, as these are unavailable in their own or nearby cities and towns. This adds to the cost of travelling and the accommodation for the stay of accompanied persons. Some of the caregiver participants mentioned that they request to stay at their family or friends' houses in that city if they remain there for days or weeks, and that this reduces the cost.

#### 3.4.2. Military Hospitals

Older care recipients eligible for military hospitals prefer these hospitals over other government or private hospitals in Pakistan. Military hospitals, such as the Combined Military Hospital (CMH) and the Pakistan Air Force (PAF), are famous and have good reputations. The caregivers and care recipients were more willing to use military hospitals' services than government hospitals.

*“I always took him to CMH as we do not have to pay much there because he was in the army. CMH is really good. All his treatments have been done there. He was even hospitalised there for many days due to paralysis.”* (FGD 1: Caregiver: Wife, Urban)

*“I took him to PAF hospital. It is far, but I had to take him there as he is much better now due to his treatment from there, so I took him there. Because of his paralysis now, his one hand does not work. I went to the PAF hospital on a rickshaw, so it takes 100 rupees on one side. It is costly to go there.”* (I4: Caregiver: Wife, Urban)

#### 3.4.3. Financial Status

Financial status is one of the main determinants of whether to use private healthcare services for both rural and urban samples. Those with low incomes use government healthcare services. However, participants showed a willingness and preference for private hospitals. Transportation costs increase the burden for lower- and middle-class families. When asked about the healthcare system's cost, all the caregivers felt that transportation is also an added cost in accessing expensive healthcare and medicines.

### 3.5. Medication Practices

#### 3.5.1. Medication Management

Women caregivers self-reported their roles included administering multiple medications on time (sometimes against the care recipient's will), sometimes having to make judgements regarding when to withhold, increase, decrease or discontinue medication, and maintaining continuous filling of prescriptions, which could involve keeping up to date with multiple prescriptions from multiple prescribers. Most caregivers mentioned that they have learnt about their regular medication and symptoms. The older people receiving care took various medicines for health issues such as diabetes, high blood pressure, arthritis, depression, asthma, respiratory conditions, chronic pain and many other long-term health conditions. Some used sleeping pills for sleeping issues. Most care receivers liked to take medicines and visit health professionals regularly (some were more reluctant). Most caregivers stated that they use allopathic medicine for acute and chronic conditions in older people's treatment and had not used homoeopathic or herbal medicines. However, a few caregivers reported that the older people under their care had used homoeopathic and herbal medicines as they used to go to the Hakeem (herbalist in the local language) in the past, and some of them are still using allopathic and herbal medicines simultaneously.

“*My father-in-law believes in herbal medicines a lot. He knows a lot about medicine and likes to take medication a lot; he also uses Hakeem's medicine. He does “Dam” [an act of puffing on self or someone after reciting spiritual versus] by himself as many people come to him for Dam as well. He Dams himself a lot.*” (I2: Caregiver: Daughter-in-law, Urban)

*“She was taking Hakeem's medicine, but it does not have any effect on her health condition. She was also consuming homoeopathic medicine, but there was no effect on her. Nowadays, she is taking medicines from the doctor.”* (I4: Caregiver: Daughter-in-law, Rural)

Most caregivers reported that care receivers stopped taking homoeopathic or herbal medicine in the past and started using allopathic medicines due to their effectiveness.

#### 3.5.2. Medication Adherence

Most caregivers showed their understanding of the importance of medicine adherence. Similarly, results of rural and urban samples suggested that the majority of the caregivers claimed that missing and delaying medicine doses negatively impacted older people's health conditions and led to their suffering. Some rural and urban caregivers claim that older people often remind them about the timing of the medicine dose and the dose.

*“My mother also reminds me to give her medicine; she remembers herself too.”* (I2: Caregiver: Daughter, Rural).

A few of the caregivers also explained the difficulties they faced while administering medicine to the care receivers, such as the unwillingness of the older people to take medicine.

*“He resists like a child and says I do not want to go to the doctor, do not take me. He will not take medicine, so I do not give it to him as I do not want to force him, and if I force him to have medicine, then he hits me. He hits as he has a stick with him, so he hits with that as he does not know whom he is hitting, how, or why he is hitting. He does not know when he gets angry. This means if I became stubborn and insisted on him taking his medicine, if I said twice or thrice, he would take his stick out to hit me.”* (I8: Caregiver: Wife, Urban).

This study showed notable medication adherence among care receivers. Most of the caregivers mentioned that a visit to the doctor and medicine are expensive. Therefore, adhering to instructions will save their efforts and money. Caregivers also reported high medication adherence among older people who were previously using different treatments, such as homoeopathic and herbal treatments. Caregivers reported that care receivers trusted allopathic medicines and switched to them due to their effectiveness and because they provided quick relief.

#### 3.5.3. Self-Medication Practices

Most of the caregivers reported self-medicating the older people under their care because sometimes they self-identify the symptoms and want to make them feel better because they do not like to see them in pain. Caregivers reported practising purchasing medicines from a pharmacy using an old prescription or sharing medicines among family with others with similar symptoms. Most caregivers said they give Panadol, Disprin and Ibuprofen for fever, body pains and cough syrups for cough and congestion to older people under their care. Some caregivers mentioned purchasing medicine from a nearby pharmacy or using leftover medicines, such as Augmentin (an antibiotic).

“*I also keep medicine, like Panadol® and Brufen® at home all the time. I give him Panadol® or Disprin® if he has a headache or if he says I have body aches, then I give him Panadol.*” (I5: Caregiver: Wife, Urban)

Most caregivers claim that they have learned from years of experience in caregiving and know which medicines work better on the older people under their care and when and which medicine to give them.

“*Sometimes, when we cannot go to the doctor and his (care receiver) medicine is finished, I use the old prescription of the doctor or show the medicines' packages to the pharmacy to get the medicines as I know the doctor will give him the same medicines again and again.*” (I9: Caregiver: Daughter, Rural)

There are many reasons found for self-medication, such as being unable to access the doctor, financial difficulties, family size, difficulty in getting an appointment, transport costs, living far from healthcare services and desire for instant relief as the pain is unbearable.

A few of the caregivers said that older people sometimes overdose and self-medicate in the case of unbearable pain and suffering and that some of them like to take medicines without any reason. The main reasons for self-medication by older people themselves were easy access to medicines, as they kept medicines near their beds or in their drawers. However, caregivers reported taking actions to avoid self-medication by older people by hiding the medicine or reducing access to it by locking the medicine in the cupboard.

“*Initially, he was taking medicine by himself, but now I give him his medicine in his hand, and then he swallows it. He was initially taking medicine as some antidepressant medicine was prescribed to him, which he used to take himself, and I thought he became addicted to it. He started thinking that if I took medicine, then I would be fine. If I sometimes forgot or got late, then he got angry that I did not give him medicine and that I did not want him to get well and wanted to kill him. All the time, he insisted on having medicine and says I wanted medicine, give me medicine.*” (FGD 1: Caregiver: Daughter-in-law, Urban)

“*She likes taking medicine. She usually says that maybe I get relief by taking more medicine, bring me this or that medicine too. She usually says give me one dose from your medicine. I usually say that if it causes a severe problem, then what will happen? My medicine is for a different disease; how can you be relieved with this? But then she insists, saying, if you can get relief from this, I will get relief too.*” (I6: Caregiver: Daughter-in-law, Rural)

Most rural and urban caregivers believed in the effectiveness of allopathic medicines because they quickly relieved pain and suffering. They always preferred to give allopathic medicines to care receivers instead of homoeopathic and herbal medicines.

#### 3.5.4. Medicine Storage at Home

All the caregiver participants said they are responsible for storing medicine for older people. The urban caregivers reported storing medicines on bedside tables, drawers, cupboards and other places near the care receivers. Therefore, when caregivers give medicine to older people, they have easy access to it and avoid mixing it with other household medicines. However, the medicines were placed openly near care receivers in rural areas. No proper care was given to prevent children from accessing the medicines or to avoid mixing these up with other medicines in the household.

“*I placed all his medicines in a drawer near his bed so when I have to give him a dose, I will easily find them and not mix them with my own or others' medicine in the home.*” (FGD 2: Caregiver: Wife, Urban)

“*I put her (mother-in-law) medicines in her cupboard, so in this way, I can keep all her medicines in one place; otherwise, sometimes she throws her medicines in anger. Then, I must find or repurchase the medicines.*” (FGD 1: Daughter-in-law: Rural)

Most caregivers in rural and urban settings were unaware of the proper storage requirements for different medicines. All kinds of care receivers' medicines were stored together in one place, for example, drawers, bedside tables or cupboards. Most caregivers from rural and urban areas reported that if the doctor instructed them about medicine storage, they remembered it and followed the instructions. Most caregivers reported storing the medicines for the older person's future use or sharing them with family members.

#### 3.5.5. Medicine Identification by Caregivers

Most of the urban caregivers were the wives of the older care receivers and could read the medicine name, prescription and instructions. Due to age and low literacy, some spouse caregivers had difficulty identifying the right medicine. However, they were usually helped by other family members, such as a son, daughter-in-law, daughter and grandchildren. Most of the caregivers in rural areas had limited education. They could not read the medicine names, instructions and prescriptions and used their ways to identify older people's medicines under their care. Most of them used the colour and packaging of medicine for identification. They also claimed that with years of experience in caregiving, they had learnt about many medicines. Some participants in rural areas sought help with medication from other family members who could read.

“*I took out one pill from each packet. They do not write anything on the medicine; I am not educated and unable to read. I remember now, as my in-laws have lived with me since my wedding. Sometimes they(in-laws) are fine and sometimes they are sick, and I have to take care of them. I took them to doctors whenever they got sick, and now my mind has all this information, such as how to give this medicine and how to do this.*” (I1: Daughter-in-law, Rural)

*“He takes medicines for blood [blood pressure] and breathing [asthma]. I gave him medicines. I remember the medicine from the colour as one is red, one is green. The pink one is for breathing. The red one is for a cough, the yellow is for allergy, and the other is for energy.”* (FGD 1: Caregiver: Wife, Rural)

*“I know about his blood pressure medicine and all other medicines. I know the medicine by seeing them as one doctor helped me understand that this is for the morning, this is for the afternoon, this is for the evening, this is for blood pressure, this is for sleep, this is for paralysis, and then I understand it. I take care of his medicine.”* (I10: Caregiver: Wife, Urban)

All the caregivers felt confident about their medication practices for the older people under their care. Most urban and rural caregivers mentioned taking medication advice from a healthcare professional in the family, such as children, grandchildren or other relatives.

## 4. Discussion

The findings of this study show the involvement of family caregivers, suggesting that women are the primary caregivers for older family members who need care. Most of the women in the family who played the role of caregiver were the daughter-in-law, spouse or daughter of the care receiver, who provided daily life assistance, including medication practices and decision-making on healthcare seeking and utilisation, consistent with previous literature [35]. Similarly, Ahmad investigated informal caregivers' experiences and found that women, particularly daughters-in-law, took on most of the caregiving role compared to men. The findings of this study provide insight into women caregivers' significant role in medication management and in seeking healthcare for older people under their care. Some rural and urban participants mentioned that the care receivers also participate in decision-making depending on their health and mental conditions, similar to other studies' findings [36]. Most of the women caregiver participants of our study reported playing an important role in decision-making, supported by the present findings, as some women caregivers play the role of principal decision-maker. However, a similar recent study found that women family caregivers (daughters) often had to make quick medical decisions on the spot as primary caregivers [37]. This is because they were assumed and expected to be solely responsible for the older people's caring responsibilities [37].

This contrasts with an earlier study by Hussain et al., who found that men were the primary decision-makers for health-seeking in Pakistan [38]. However, our findings suggest that sometimes other family members, such as children, spouses or relatives of the care receivers (depending upon the family system, such as a nuclear or joint family system), are also involved in decision-making, which aligns with other studies' findings [5, 39].

Our findings suggested that many factors, such as financial restraints, consistent with the prior literature, cause delays in healthcare access [40]. Low-income levels and large family sizes align with earlier studies conducted in Pakistan [40, 41]. Most of the rural caregivers mentioned that the cost of travelling was in addition to the cost of healthcare. The rural caregivers showed more delay in accessing healthcare than urban caregivers for older care recipients. Our study also highlighted that the urban population of older care recipients is more eligible for healthcare services, as they are more educated and mostly retired military or government servants, compared to rural care recipients, who usually work as farmers with limited education. It is also important to note that those eligible for military hospital services appropriately utilised healthcare services in the urban sample. However, the rural sample was not appropriately using healthcare services because of the distance, and the transport doubled their cost. The distance to healthcare facilities was also considered a barrier, as the rural caregivers mentioned, and transport was the first and foremost hurdle in accessing appropriate healthcare for older people. We found that distance and transportation costs are significant barriers for rural caregivers, impacting healthcare utilisation for older persons under their care. Similarly, previous literature in Pakistan finds transportation to be a barrier to healthcare access [38, 42].

Most rural and urban caregivers in our study prefer private healthcare, but the cost remains a significant barrier. However, multiple studies have shown that this preference is linked to private hospitals' access to life-saving equipment, shorter waiting times, more convenient hours and better perceived service quality [41]. In contrast, chronic staff and medicine shortages in public/government hospitals deter use. Comparative satisfaction studies show higher scores for private hospitals for cleanliness, responsiveness and timeliness [43].

Many other factors affected self-medication and seeking healthcare for care receivers, such as treating at home, using home remedies and the availability or easy access to medicines for self-medication. This study highlights that medication practices and healthcare access for older people in Pakistan start with treatment at home for minor to significant symptoms. This study suggests that all the caregivers and recipients believe that allopathic medicines' quick relief and effectiveness were widespread in rural and urban samples. These findings are consistent with results from previous studies conducted in Pakistan [44–46]. The women caregivers also mentioned storing medicines for future use. This study's results are consistent with previous research in this area [47].

Some medicines sold without a prescription by unqualified persons in medical stores and dispensaries can be dangerous. In Pakistan, most pharmacy workers have minimal formal education, with 10–12 years of schooling and with little or no professional training in the pharmacy/medical field [48]. As a result, many pharmacies operate without a pharmacist present [48]. Medicines can cause adverse effects on older people, and self-medication can pose a high risk for this population, especially if they are unaware of indications and interactions. Both international literature and the limited local studies on self-medication for older people highlight the risk associated with self-medication among this population [49–52]. A visit to a nearby health dispensary is also often reported in the rural sample. Most of the caregivers in the rural and urban samples are not aware of the danger of self-medication among older people or the proper storage of medicine and child safety from the medicines at home. Caregivers were responsible for managing older people's medication practices and preventing excessive medication intake.

The literature suggests that medication adherence is more challenging and becomes more significant if the informal caregivers (not the patient) are responsible for medication administration and management [53–56]. In our study, most caregivers reported practising medication adherence for the older people under their care. Baig et al. reported that almost one-third of older patients are incapable of taking their medicines regularly, mostly because of their meagre socioeconomic status, forgetfulness and medicines they consider unnecessary [36]. However, all the participants in the current study reported medication adherence, which is contrary to the results of a prior study conducted in Pakistan by Saqlian et al. They examined medication adherence and its influences among older Pakistani hypertensive patients. The study found that low health literacy is linked to poor medication adherence among older hypertensive patients [23]. They concluded that the capability to perform ADLs is another significant aspect that positively accompanies medication adherence.

Healthcare workers can deliver caregiver education in community settings or by nurses at discharge and first OPD contact, reinforced by pharmacists' education, which should cover indications, dosing, adherence tools and safe medicine storage. We recommend policy actions to address barriers, such as distance, rurality and out-of-pocket costs, including basic OPD coverage for older adults' consultations/tests/essential medicines, transport support and rural outreach/teleconsultations to improve timely care.

The study has several limitations. A purposive sampling technique was employed, which may not fully represent the broader population of caregivers for older people in Pakistan, such as male caregivers. Additionally, the study was confined to one district's rural and urban areas, limiting the findings' generalisability to other districts. The reliance on self-reported data from caregivers also introduces potential biases, such as social desirability or recall bias, which may have influenced the results.

The current study adds a qualitative dimension to the family caregiving literature in Pakistan by examining geriatric care provided in homes. It also provides future research directions, particularly to explore the impacts on caregivers' health. In addition, this study focussed primarily on women; exploring men's perspectives towards caregiving would be beneficial since their experiences may differ due to their sociocultural and economic positions.

## 5. Conclusion

Women caregivers play a significant role in decision-making regarding seeking healthcare and medication practices. However, they operate under financial and geographic constraints, which may cause delays in accessing health services, caring for patients at home and self-medication during the initial stages of illness. Implementing concise, actionable training alongside targeted policies on OPD coverage, transport and medicine safety is likely to improve timely and safer care for older people.

## Figures and Tables

**Table 1:** Demographic characteristics of focus group and individual interview participants.

	Focus group		Interviews	
	Rural	Urban	Rural	Urban
	*n* = 15	*n* = 17	*n* = 10	*n* = 10
Age range caregivers (years)				
20–39	3	4	2	3
40–59	8	6	5	3
60–79	4	7	3	4
Age range care receiver (years)				
60–69	0	0	0	1
70–79	4	7	3	4
80–89	11	10	7	5
Education				
Uneducated	4	0	3	0
Primary	6	0	1	0
Middle	3	3	1	0
Secondary	2	5	4	3
Tertiary	0	9	0	7
Occupation				
Housewife	5	13	4	5
Casual work	10	0	4	1
Full-time work	0	4	2	4
Relationship to care receiver				
Daughter	2	4	2	2
Daughter-in-law	9	6	5	4
Wife	4	7	3	4

## Data Availability

The data that support the findings of this study are available upon request from the corresponding author. The data are not publicly available due to privacy or ethical restrictions.
